# The Influence of Cisplatin on Functionality and Surface Characteristics of Mesenchymal Stromal Cells In Vitro

**DOI:** 10.3390/ijms27010076

**Published:** 2025-12-21

**Authors:** Armin von Fournier, Totta Ehret Kasemo, Miguel Goncalves, Stephan Hackenberg, Marietta Herrmann, Marianne Schmidt, Manuel Stöth, Till Meyer, Thomas Gehrke, Agmal Scherzad

**Affiliations:** 1 Department of Oto-Rhino-Laryngology, Head and Neck Surgery, University Hospital Wuerzburg, 97080 Wuerzburg, Germany; 2 IZKF Group Tissue Regeneration in Musculoskeletal Diseases, University Hospital Wuerzburg, 97074 Wuerzburg, Germany; 3 Bernhard-Heine-Centrum for Locomotion Research, Julius-Maximilians-Universitat Wuerzburg, 97074 Wuerzburg, Germany

**Keywords:** cisplatin, MSCs, mesenchymal stromal cells, stem cell characteristics, differentiation, migration, functionality

## Abstract

Mesenchymal stromal cells (MSCs) are multipotent and play an important role in regenerative processes such as wound healing. Data on possible changes and functional restrictions of MSCs due to cisplatin chemotherapy, for example, in the treatment of head and neck cancer, diverge. The aim of this study was to evaluate the influence of cisplatin on MSCs with regard to their defining characteristics and their ability to differentiate and to migrate. MSCs from four human donors (a 59-year-old man, a 63-year-old woman, a 70-year-old man, and a 61-year-old man) were cultured in vitro with and without cisplatin for 24 h, and toxic and subcytotoxic concentrations were determined using an MTT. We then examined the surface phenotype markers (flow cytometry), migration (scratch assay), histological differentiation markers (adipo-, chondro-, osteogenic), and the expression of selected line-associated genes in real-time quantitative PCR (RT-qPCR) (LEP, SOX9, RUNX2). These characteristics were evaluated after treatment with different subcytotoxic, clinically relevant doses of cisplatin. Flow cytometry confirmed the presence of MSCs-characteristic surface markers, which remained stable under treatment with subcytotoxic doses of cisplatin. Cisplatin exposure reduced the mRNA abundance of leptin (a marker for adipogenic differentiation) but increased SOX9 mRNA abundance (chondrogenic differentiation). RUNX (osteogenic differentiation) did not change post cisplatin exposure. Histological analysis showed no difference with regard to osteogenic, chondrogenic, and adipogenic differentiation at doses up to 10 μM cisplatin. Cell migration was not restricted by cisplatin exposure under the conditions used here. The characteristics of MSCs were not different to controls post cisplatin exposure. mRNA analysis suggested induced changes by cisplatin, although this effect was not histologically detectable even at high doses. Based on the single-molecule markers used here, indications for an inhibitory effect of cisplatin on adipogenic differentiation and a rather enhancing effect on chondrogenic and osteogenic differentiation may be hypothesized. The process observed here could further aggravate the already serious problem of malnutrition in head and neck cancer patients, for example. Taken together though, our study confirms overall MSCs tolerance towards cisplatin.

## 1. Introduction

Mesenchymal stem cells (MSCs) are multipotent cells with features which are promising in, e.g., tissue engineering and other medical research, but may also play so far poorly understood roles in manipulating, for instance, tumor microenvironments. Cisplatin is a commonly used chemotherapeutic compound. Understanding potential interactions between chemotherapeutics and multipotent cells is crucial in order to avoid patient harm and optimize therapeutic success.

MSCs is a frequently used term for multipotent progenitor cells, which can be isolated particularly from bone marrow [1], but also from other tissue types such as cartilage or fat [2,3]. These cells can differentiate into various functional cell types. A distinction is often made between osteogenic, chondrogenic, and adipogenic differentiation [4]. Differentiation of MSCs can be specifically induced in vitro [5,6].

Study results on mesenchymal stem cells (MSCs) were previously difficult to compare due to differing methodologies. To overcome this limitation, the “Mesenchymal and Tissue Stem Cell Committee of the International Society for Cellular Therapy” established criteria to define human MSCs as uniformly as possible [5].

Functions of MSCs in the human organism are diverse and continue to be the subject of intensive research [4,7]. A clear role in the regeneration of body tissues such as bone, cartilage, fat, and connective tissue, as well as in the development of blood progenitor cells, has achieved consensus [8]. Furthermore, MSCs secrete proteins, e.g., for the formation of the extracellular matrix [9,10], and immunomodulatory properties have been demonstrated [11]. The latter are also of great importance in malignant tumors, where MSCs within the tumor microenvironment influence tumor biology and immunology through the release of cytokines, chemokines, and growth factors [12].

MSCs are also finding clinical applications in, e.g., stem cell transplantation after radiation or chemotherapy, or in the field of tissue engineering.

Cis-diamminedichloroplatinum (II) (cisplatin) is one of the most important chemotherapeutic agents used as a cytostatic agent for the treatment of numerous malignant human tumors. The primary mechanism of action is apoptosis induction through DNA cross-linking and mitosis inhibition [13]. Various studies also point to additional mechanisms of action that could lead to other partly undesirable but also partly potentially beneficial side effects. One example is cisplatin-induced immune modulation [14,15]. Among other things, the impairment of MSCs’ function in peritumoral biological processes is also under discussion. In cisplatin-treated mice, a significant decrease in myeloid-derived suppressor cells (MDSCs) was observed in tumor-draining lymph nodes (TDLNs) [16], clearly demonstrating additional effects of cisplatin on MSCs.

Among potential side effects, the influence of cisplatin on one of the key functional characteristics of MSCs, and their ability to differentiate between them, remains unclear. Despite the potentially high relevance of this topic, our literature search revealed only two studies on this topic, each of which reported conflicting results [17,18].

The aim of this study is therefore to investigate the influence of cisplatin on donor-derived MSCs beyond mere toxicity, namely with regard to a possible alteration of their characteristic properties and functionality. To this end, their ability to differentiate, and the expression of, characteristic surface markers was investigated. Furthermore, the functionality of the cells with regard to their migration capacity was evaluated.

## 2. Results

### 2.1. Defining Toxicity and Subcytotoxic Ranges of Cisplatin on MSCs

**Figure 1:**
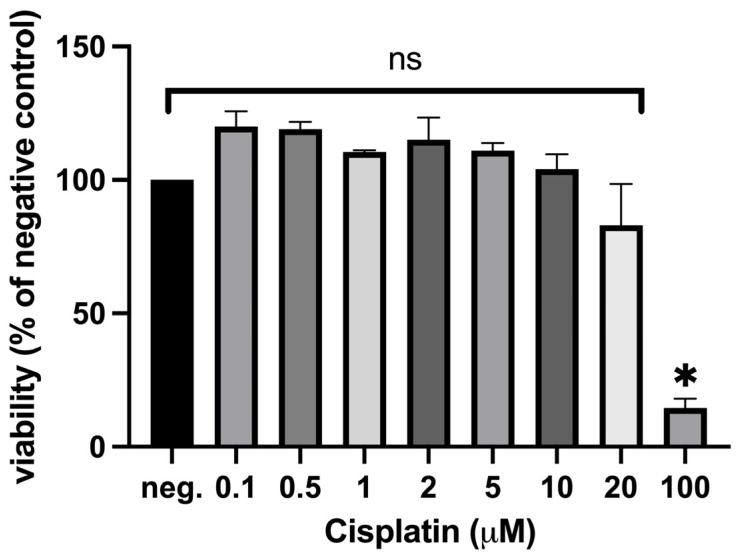
Cytotoxicity of cisplatin on MSCs in the MTT assay as measured by the relative viability compared to the negative control (neg., 0 μM). A significant reduction in viability was detected at 100 μM; Donors 2, 3, and 4 were included (n = 3); data is presented as mean value ± SEM; ns = non-significant result (one-way ANOVA) and asterisk indicates statistically significant result (*p* ≤ 0.05).

In order to determine suitable conditions to evaluate the effects of cisplatin on MSCs differentiation, MSCs from three donors were treated with increasing concentrations of cisplatin (0.1 μM, 0.5 μM, 1 μM, 2 μM, 5 μM, 10 μM, 20 μM, and 100 μM), and viability was determined in a metabolic assay (MTT assay). Cisplatin showed a cytotoxic effect on MSCs at 100 μM, with a sub-toxic concentration range up to 20 µM, although from >10 μM a not yet significant reduction trend in viability was observed (Figure 1). At low cisplatin concentrations, viability exceeded the negative control, as is often observed in metabolic activity assays (see Section 3).

### 2.2. Analysis of Surface Markers at Different Cisplatin Concentrations

**Figure 2:**
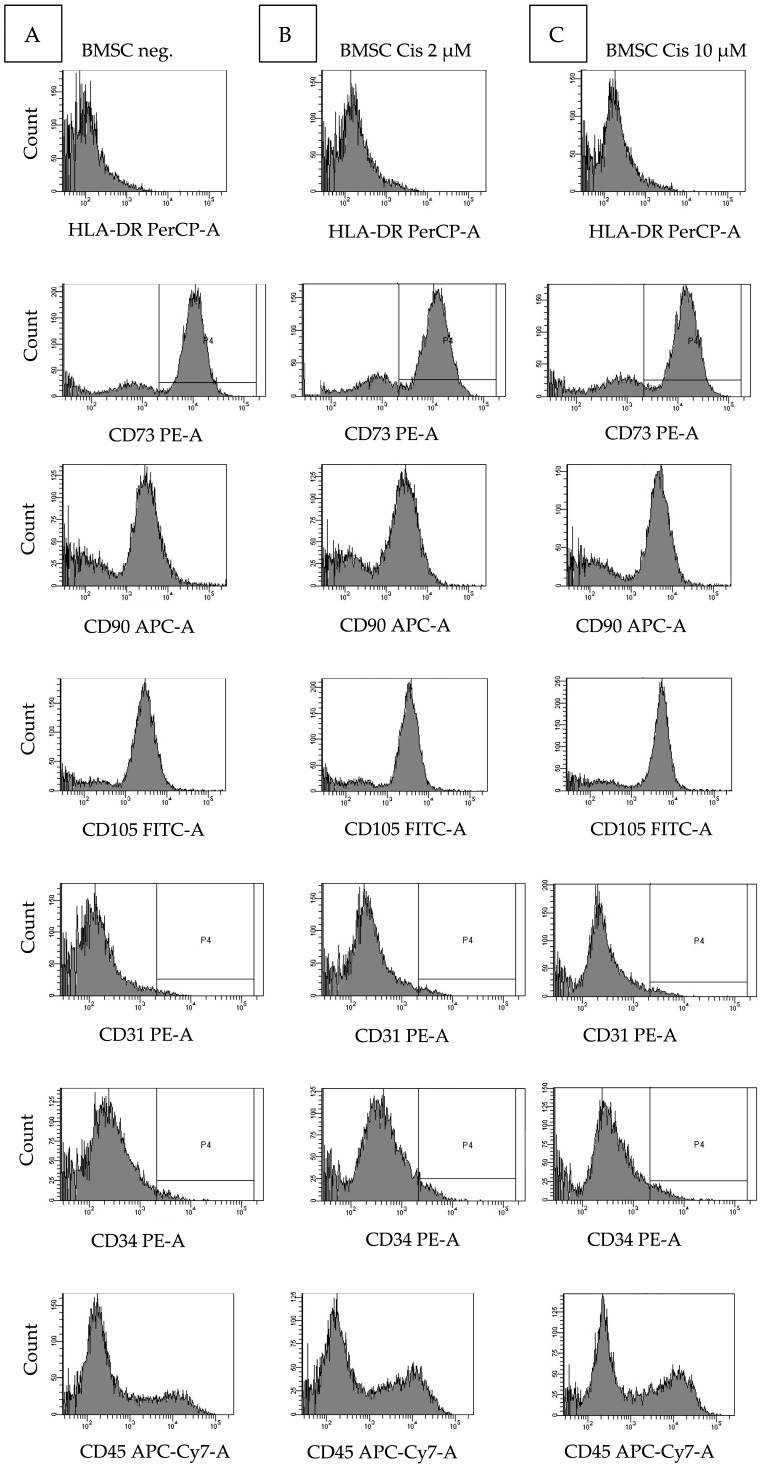
MSCs-typical constellation of six surface antigens was evaluated by flow cytometry with and without cisplatin. Donors 2, 3, and 4 were included (n = 3). Here, constellation of one donor (Donor 2) is shown. There is no impairment after cisplatin treatment. Negativity for CD31, CD34, and CD45, positivity for CD73, CD90, and CD105. Column (**A**) Surface markers without cisplatin treatment. Column (**B**) With 2 μM cisplatin. Column (**C**) With 10 μM cisplatin.

Cells were examined for the stromal cell-typical constellation of surface antigens using flow cytometry. Surface antigens CD73, CD90, and CD105 were detected, whereas CD31, CD34, and CD45 were not detected on the surface of cells. The procedure was repeated after treatment with 2 μM cisplatin. No impairment of the surface features was detectable post cisplatin exposure. These results were reproducible for three donors (Figure 2).

### 2.3. No Cisplatin-Induced Changes in MSCs Differentiation by Histological Determination

**Figure 3:**
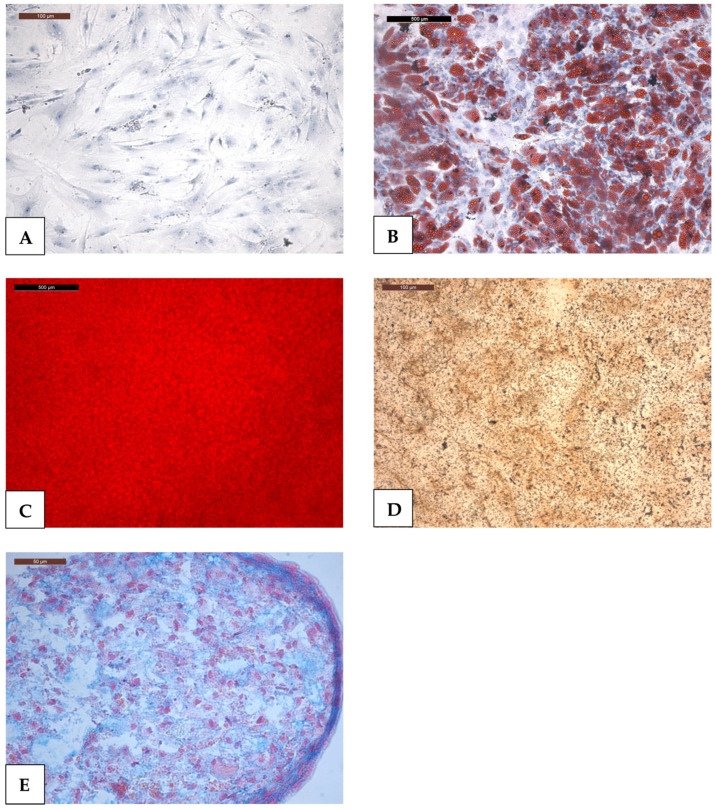
Histological staining after differentiation confirmed the presence of adipogenic, osteogenic, and chondrogenic phenotypes in MSCs from two donors. Donors 1 and 3 were included (n = 2). The example images shown here are from Donor 1; (**A**) MSCs native in DMEM-EM; (**B**) red fat vacuoles in the Oil Red O staining (adipogenic differentiation); (**C**) red–orange deposits in the Alizarin Red S staining and (**D**) brown–black calcium deposits in the Von Kossa staining (osteogenic differentiation); (**E**) blue acidic mucopolysaccharides in Alcian Blue staining (chondrogenic differentiation).

To first test the identity of the cells isolated and treated in medium for differentiation into a specific MSCs phenotype (osteogenic, chondrogenic, and adipogenic), cells were stained using three different methods. The presence of appropriate differentiation was evaluated. The stains used confirmed the presence of adipogenic, osteogenic, and chondrogenic phenotypes (Figure 3). The procedure was repeated after treatment with cisplatin doses of 2.5 μM, 5 μM, and 10 μM. No differences in the staining pattern were observed after cisplatin treatment. Since only two donors were used for histological staining, statistical analysis would not be reliable. This is also due to the limited number of donors and, therefore, donor stem cells available to us. In addition, digital color intensity analysis is not applicable in this experiment. We used this descriptive experiment as a supplement to the statistically evaluated ones. Additional images can be provided upon request. However, as already described, no effect of cisplatin pretreatment on staining was observed here.

### 2.4. Detection of Differentiation by Analysis of Gene Expression (RT-qPCR) Under Different Concentrations of Cisplatin

**Figure 4:**
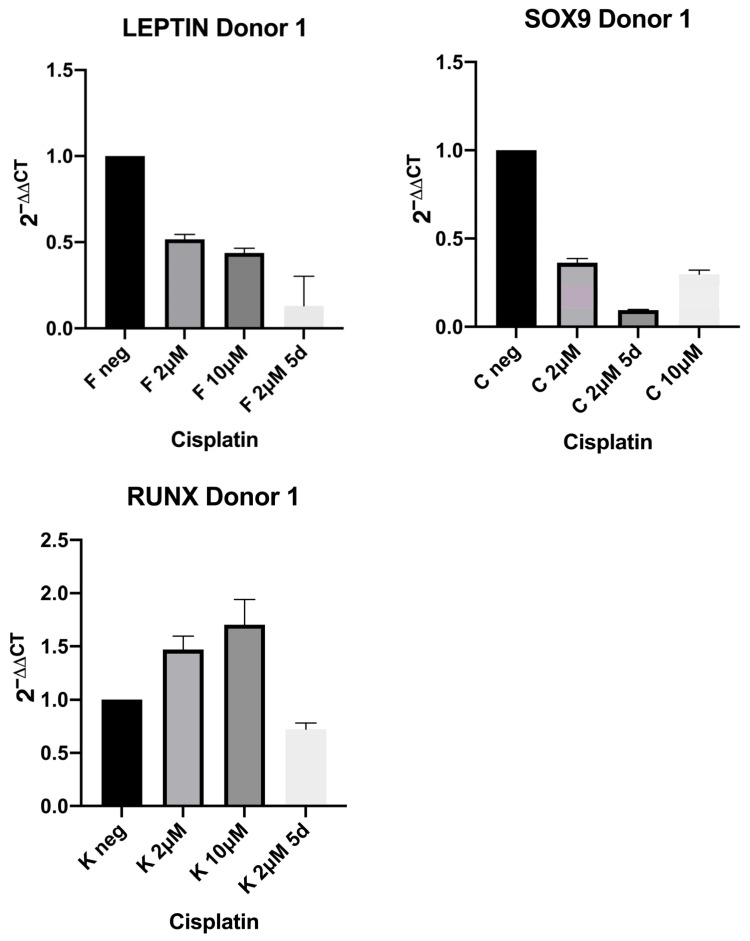
In Donor 1, the expression of Leptin, SOX9, and RunX in RT-qPCR was evaluated untreated and after treatment with cisplatin doses of 2 μM, 10 μM, and 2 μM for 5 days. A decrease in leptin and SOX9 expression can be observed after treatment with cisplatin. For RunX expression, an increase in expression is observed. In all three target genes, a further decrease in expression was observed after prolonged treatment with cisplatin for 5 days; data is presented as standard error (mean ± SEM) of technical replicates from Donor 1.

**Figure 5:**
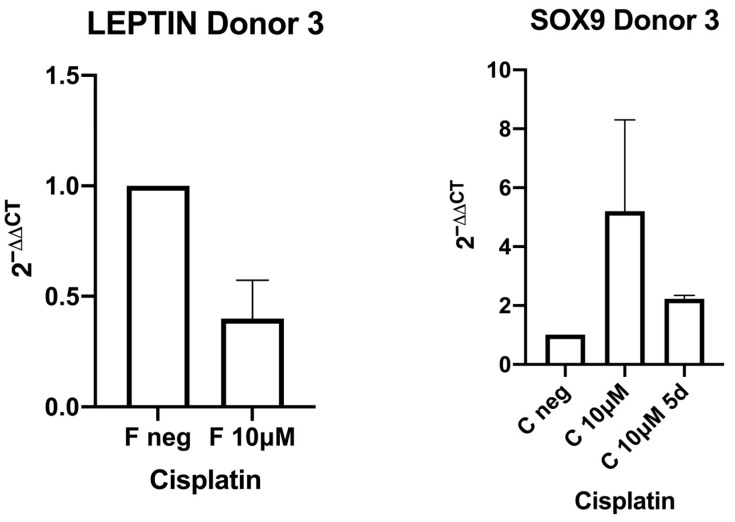
In Donor 3, the expression of Leptin and SOX9 in RT-qPCR was evaluated untreated and after treatment with a cisplatin dose of 10 μM, and for SOX9 additionally 10 μM for 5 days. A decrease in leptin and increase in SOX9 expression can be observed after treatment with cisplatin. For SOX9, a further decrease in expression was observed after prolonged treatment with cisplatin for 5 days; data is presented as standard error (mean ± SEM).

To further investigate the influence of cisplatin on differentiation (osteogenic, chondrogenic, and adipogenic), the expression of leptin (adipogenic differentiation), SOX9 (chondrogenic differentiation), and RunX (osteogenic differentiation) was investigated using RT-qPCR (Figure 4 and Figure 5). The data in the figures are presented as standard error (mean ± SEM) because they are data from three technical replicates each, which were carried out to achieve increased precision and the exclusion of measurement errors. The expression was evaluated untreated and, in one donor, after treatment with cisplatin doses of 2 μM, 10 μM, and 2 μM for 5 days. In the second donor, the expression was evaluated untreated and after exposure to a dose of 10 μM cisplatin and after 10 μM for 5 days. In both donors, a congruent decrease in leptin expression was observed after treatment with cisplatin. In the donor examined for RunX expression, an increase in expression was observed. For SOX9 (both donors were examined), diverging results were observed. In one donor, a decrease in expression is evident, and in the other donor an increase is evident. In every case examined, regardless of the target gene, a further decrease in expression was observed after prolonged treatment with cisplatin for 5 days. Because of the limited number of donor stem cells, the results with an additional 5-day exposure time could only be acquired from a single donor. This descriptive experiment can be considered a supplementary addition. 

### 2.5. Migration Analysis with the Scratch Assay

**Figure 6:**
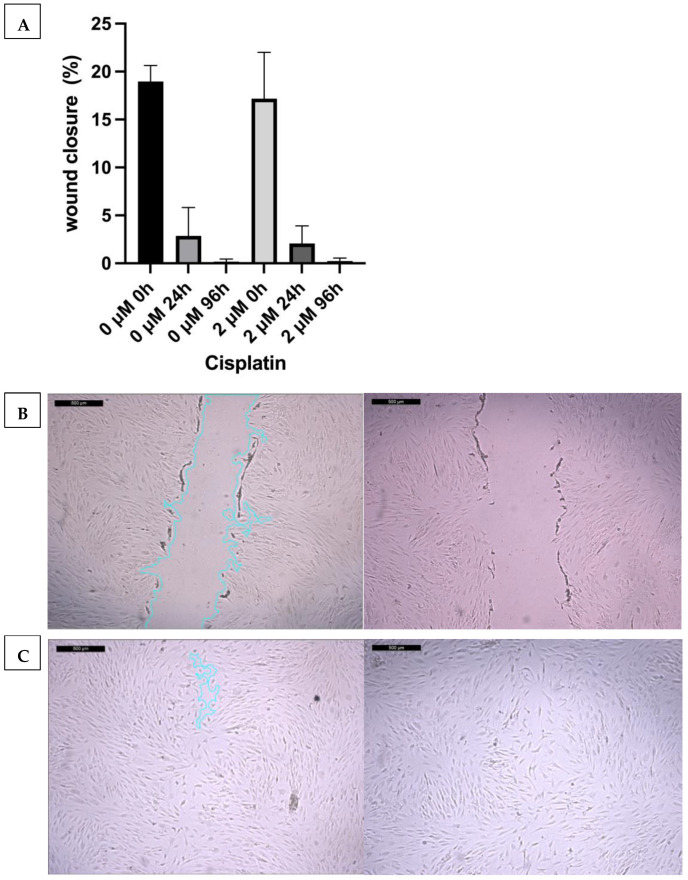
No significant change in migration after treatment with cisplatin (one-way ANOVA); data is presented as standard error of the mean (mean ± SEM) (**A**). Migration progress in the scratch assay (here representative images without (**left**) and with (**right**) prior cisplatin treatment) at 0 h (**B**) and 24 h (**C**). 0 h: picture straight after adding the cell-free aisle. 24 h: almost completely closed aisle as a sign of wound closure. Donors 2, 3, and 4 were included (n = 3).

In the scratch assay, migration ability of MSCs after treatment with cisplatin was analyzed. Here, a cell-free lane (“scratch”) is created in the cell culture. The speed at which this “scratch,” which represents a wound, closes is documented. Wound closure is observed at 0 h, 24 h, and 96 h and then compared. No significant difference in the wound closure speed was observed after treatment with 2 μM cisplatin. The scratch was almost completely closed after 24 h and completely closed after 96 h, both with and without prior treatment with cisplatin (Figure 6). This argues against a change in migration ability under the subcytotoxic cisplatin dose used.

## 3. Discussion

The aim of this study was to evaluate the influence of cisplatin on MSCs with regard to their defining characteristics, their differentiation capacity, and their functionality with regard to migration capacity.

Cisplatin is highly relevant as a widely used chemotherapeutic agent in tumor therapy. MSCs too are central in many biological processes such as regenerative processes and, as recently described, in peritumoral processes, such as in the tumor microenvironment. There, MSCs can influence tumor immunology through the release of cytokines, chemokines, and growth factors [19,20]. Since off-target effects have been documented for cisplatin and this is a relatively poorly investigated field, the authors were motivated to investigate the influence of cisplatin on MSCs.

As expected, cisplatin demonstrated concentration-dependent cytotoxicity in MSCs. Interestingly, low doses increased metabolic activities above control levels. We often observe higher values at lower dose concentrations than the negative control in the MTT assay, as was the case here with cisplatin. This is probably due to the fact that this is a metabolic activity assay. Perhaps cells react to the substance and, for example, try to remove it by increased activity, possibly reflecting hormesis or cellular stress responses. Molecular characteristics of MSCs were demonstrated, as is typical in stem cell studies. In addition, these criteria were evaluated after treatment with sub-toxic cisplatin concentrations for 24 h, revealing no change in the characteristic surface features. Multipotency was demonstrated at the mRNA-level using RT-qPCR. Interestingly, the results indicate a compromise of adipogenic differentiation (decrease in leptin expression) after treatment with cisplatin. Meanwhile, osteogenic differentiation appears unaffected or even enhanced. The results for chondrogenic differentiation are ambiguous or divergent with differences between donors. Histologically, Von Kossa, Oil Red O, and Alcian Blue staining were performed and would indicate adipogenic, osteogenic, and chondrogenic differentiation, respectively. No changes were observed after cisplatin treatment. Similarly, no effect on migration ability was observed at subcytotoxic concentrations. Overall, the high stability of MSCs towards the antitumor drug cisplatin was confirmed. The impairment of adipogenic differentiation by cisplatin could be an important finding, as this could further aggravate the already serious problem of malnutrition in head and neck cancer patients [21,22]. In the light of anti-tumor therapy, repeated treatment and recovery phases would be relevant extensions to evaluate cisplatin influence on MSCs of longer periods of time in a mimicked therapy scheme. However, current in vitro methods for donor-derived MSCs limit such approaches, so that additional optimization of culture conditions would be required to remain in the 3R-scheme to replace, reduce, and refine animal experiments and to gain human-relevant data.

According to our literature research, despite the potentially high relevance of this topic, there have only been two studies on this topic to date, each of which came to quite different conclusions [17,18].

In one of those studies, the influence of cisplatin on the characteristics of bone marrow-derived multipotent mesenchymal-stromal cells (BM-MSCs) was investigated and discussed in several respects [18]. The authors observed a fairly high resistance of the MSCs, in line with our findings. In detail, they found that cisplatin did not affect cellular morphology or adhesion capacity. Furthermore, no apoptosis was triggered in MSCs under the selected conditions. Interestingly, the ability to differentiate the typical surface characteristics were also preserved. The authors of this study further discussed that cytoskeletal rearrangements and the expression of heat shock proteins, which were observed, could be partly responsible for the observed cisplatin resistance of the MSCs. The mechanisms underlying this resistance of MSCs to cisplatin would certainly be an interesting starting point for future experimental studies. In particular, the reasons for the continued stable expression of MSC surface markers, which remains stable even under high doses of cisplatin in the aforementioned study as well as in the present work, are of interest. A promising approach here would be to first gain a better understanding of the role of heat shock proteins in this context, which have already been shown to have a protective effect against cisplatin in tumor cells as well as in physiological cells [23,24,25].

The other study reported the influence of cisplatin on the differentiation ability of adipose tissue-derived mesenchymal stem cells (ADSCs) [17]. Here, the examination of differentiation by histological staining (Oil Red O, Alizarin Red, and Alcian blue) after cisplatin treatment showed a decrease in adipogenic and osteogenic, as well as chondrogenic, differentiation. The authors demonstrated the expression of genes used to confirm differentiation. Interestingly, in this study, the ADSCs from one donor that had been treated with cisplatin showed significantly decreased adipogenic differentiation but increased osteogenic differentiation compared with ADSCs derived from a donor who was healthy. This compares to our data, which also showed donor-specific responses. We observed a congruent decrease in adipogenic differentiation in both donors. In the donor examined for osteogenic differentiation in our study, an increase could be observed. For both adipogenic and osteogenic differentiation, a decrease in expression was observed after prolonged treatment with cisplatin for 5 days. Ultimately, for the study mentioned, it is unclear whether these different results were a cause of the previous cisplatin exposure or whether other circumstances could be causative. The authors suggested investigating the differentiation abilities of ADSCs before their transplantation for repairing cisplatin-induced tissue damage. What is particularly interesting in this study is the fact that adipogenic differentiation was compromised in all donors in the study, including the deviant donor, as in the present study. A possible influence of cisplatin on the surface characteristics of ADSCs was not investigated in this study. It must be mentioned that there has been debate about whether ADSCs and (BM-)MSCs could be intrinsically different cell types [26]. Both in vitro and in vivo in the mouse model, cell-specific differences at transcriptional and proteomic levels according to their tissue origin and also certain differences in their differentiation processes could be shown. Nevertheless, here they showed the same ability to differentiate towards chondrocytes and osteoblasts [26]. Therefore, even though the data on cisplatin effects on stem cells so far is scarce and not completely congruent, indications of (1) reduction in adipogenic differentiation potential, (2) donor-specific responses to cisplatin, and (3) possible time-dependent effects are noted. Designing studies with streamlined protocols and which specifically address these points and hurdles, as well as potentially interesting biology, is called for. Additionally, although challenging, larger donor numbers and technical replicates would significantly increase the possibility for statistical analysis and thereby strengthen possible interpretations of results.

However, the interactions between MSCs and cisplatin also appear to be more complex. It is to date not clear whether these effects can be considered predominantly beneficial or detrimental in terms of tumor therapy in particular or in terms of morbidity in general. The opposite—influences of MSCs on the effects of cisplatin—has also been observed. In a relatively recent study, more light was shed on the mechanisms underlying the amelioration of cisplatin-induced kidney injury by mesenchymal stromal cells. The authors concluded that systemic administration of MSCs can attenuate cisplatin-induced acute and chronic kidney injury by paracrine TSG-6 secretion [27]. Another study showed that triple co-cultures with BM-MSCs (bone marrow mesenchymal stem cells) + SH-SY5Y cells (human neuroblastoma cells) + PBMCs (peripheral blood mononuclear cells) were more resistant to cisplatin (CDDP) than the double (BM-MSCs + SH-SY5Y cells)- and monocultures of SH-SY5Y cells. The authors saw prospects for the development of novel test systems for anti-tumor agents [28]. In a more recent study by our own research group, a decrease in IL-6 and IDO1 at both protein and mRNA levels was shown after cisplatin treatment of MSCs. This could indicate that cisplatin might not only act via the known mechanism of cytotoxicity in tumor therapy, but also via a reduction in tumor-supporting proteins in the tumor microenvironment, in the sense of immunomodulation [15]. Interestingly, apart from this study, the influence of cisplatin on MSCs migration has not yet been specifically investigated. In the present study, the result that migration is not impaired by one sub-toxic dose with 24 h post-treatment time of cisplatin can be confirmed. The focus of this previous study, however, was on the immunomodulatory effect of cisplatin in MSCs, specifically the suppression of IL-6 and IDO1 and the associated potential effects on the tumor microenvironment. To identify these potentially immunomodulatory factors, enzyme-linked immunosorbent assay (ELISA) and RT-qPCR were performed after administration of different doses of cisplatin. These preliminary results, together with the investigation of the effects of cisplatin on the differentiation and surface characteristics of MSCs, now provide a somewhat more complete picture and contribute to pointing out useful future directions for complementary work.

A major challenge with in vitro experiments with human-derived MSCs is the current limited and variable cell yield from human donors, restricting the number of assays. This leads to an experimental situation in which cell numbers sometimes are sufficient for a certain number of assays, whereas cells from a different donor may only be sufficient for a lesser number of assays or replicates. Therefore, MSCs from all donors could not be used in every experiment. For the determination of mRNA markers of stromal cell differentiation (RT-qPCR), two donors were used. Differences and the lack of possibility for statistical analysis clearly demonstrate that a larger number of donors certainly is desirable. Furthermore, while the results from this experiment are overall congruent for the two donors in that assay, certain differences in chondrogenic differentiation were observed. Comparably, previous studies mentioned that they had failed to provide a sufficiently high number of donors [29,30]. Significant differences in one study were discussed to possibly result from previous cisplatin therapy in one of the donors, demonstrating the challenge in finding suitable and sufficient numbers of donors. Other authors may struggle with the challenge of not having access to donor data at all, and hence not being able to control for such pretreatments in analysis and interpretation. Differing results between donors are not surprising in this context: it is a well-known phenomenon that MSCs exhibit donor variability [29]. This can depend on a variety of parameters, such as the above mentioned previous therapy, sex, age, or genetics. Obtaining a sufficient number of usable cells from a single donor to generate biological and technical replicates for statistical analysis of the assays applied in this study is, to date, not realistic, especially since only adult donors are usually considered. The in vitro expansion of MSCs, in turn, generates its own disadvantages [29,31,32,33]. Therefore, improved in vitro conditions for human-derived MSCs should be a priority for this research field. Until such progress has been made, narrowing down the hypotheses and simplifying experiments to focus on a smaller number of parameters may improve the quality of data and enable statistical analyses. This study contributes to this effort by providing first indications for, e.g., changes in adipose differentiation and the lack of changes in migration.

In summary, the surface characteristics of MSCs and their migration ability qualitatively remained stable under single dose cisplatin exposure. The differentiation at the mRNA level seems to be affected by cisplatin, although this effect is not histologically visible even at high doses. Potentially, translation of these differences to the protein level are weak under the conditions used here and, e.g., repeated exposure or longer post-incubations times could reveal further reaching effects at the protein level or elsewhere. Similarly, additional assays or inclusion of more genes/proteins could shed more light on the relevance of the differences at the mRNA level. Interestingly, an inhibiting effect on adipogenic differentiation was evident, in line with comparable previous work on this topic. Furthermore, an enhancing effect on osteogenic differentiation was observed, although this was only examined in one donor. With regard to chondrogenic differentiation, controversial results emerge in the two donors included. Given the high clinical relevance of cisplatin-containing chemotherapies, the results could provide an important basis for further studies. Based on the present study, larger multi-donor cohorts with extended, sequential cisplatin dosing are needed to establish robustness and clinical relevance and to determine whether the observed transcriptional changes translate into functional effects on MSCs differentiation. The conditions and readouts defined here could ultimately inform approaches that preserve treatment efficacy while reducing treatment-related morbidity.

## 4. Materials and Methods

The MSCs used were isolated from residual acetabular reaming material obtained from patients receiving total hip arthroplasty. Donor consent of the four donors was obtained prior to surgery, and experiments with donor cells were approved by the ethics committee of the Julius Maximilian University of Würzburg, Germany (91/19-me). The following characteristics are known about the donors: Donor 1: 59 years old, male; Donor 2: 63 years old, female; Donor 3: 70 years old, male; Donor 4: 61 years old, male. Donor cells from the same donors used in this study were also used in a preliminary study by our research group on cisplatin-mediated IL-6 and IDO1 suppression in mesenchymal stromal cells [15]. In no case were the same results used; rather, all experiments presented were conducted specifically for this study.

### 4.1. Preparation of Cells

The bone spongiosa was transferred to a 50 mL centrifuge tube (GB227270-N, Greiner Bio-One GmbH, Frickenhausen, Germany) and filled with 50 mL of a mixture of equal parts Dulbecco’s Modified Eagle’s Medium (DMEM) and DMEM: Nutrient Mixture F-12 (DMEM/F12). The tube was manually shaken to detach the cells from the spongiosa and centrifuged (5810 R Eppendorf AG, Hamburg, Germany) at 1200 rpm for 5 min at room temperature (RT).

The supernatant containing cell culture medium and fat was aspirated using a membrane vacuum pump (KNF Neuberger GmbH, Freiburg, Germany), and 50 mL DMEM/F12 were added. After shaking and the sedimentation of the remains of the bone and connective tissue, the cell-rich medium was decanted to a new tube. The final supernatant containing cells was centrifuged at 1200 rpm for 5 min. The supernatant was discarded, and the remaining cell pellet was resuspended in stromal cell expansion medium (DMEM-EM; DMEM, 10% heat-inactivated fetal calf serum [FCS, BSF, Linaris, Wertheim, D], 1% penicillin/streptomycin [Biochrom AG, Berlin, Germany]). The cell number was determined in the hematocytometer. At least 8 × 10^8^ to ideally 1 × 10^9^ cells were seeded in 175 cm^2^ cell culture flasks (GB660160, Greiner Bio-One GmbH, Frickenhausen, Germany) with 27 mL DMEM-EM. Three to four days later, cells were washed with 15 mL of sterile phosphate-buffered saline (PBS, Roche Diagnostics, Mannheim, Germany) and refilled with 21 mL fresh medium. After reseeding and trypsinization, the cells were used for the experiments. Trypsin was inhibited by the addition of DMEM with 10% FCS. To determine the number of cells and cell viability by means of triple measurement with the cell counter CASY^®^ (Innovatis, Reutlingen, Germany), 10 mL of CASYton (isotonic saline solution, Innovatis, Reutlingen, Germany) were placed in a cell counter vessel (CASY-Cups Innovatis, Reutlingen, Germany). 10 μL of the trypsinated cell suspension was added and mixed.

The cells were cultured and at an estimated 80% confluency, the cells were passaged by aspirating the medium and washing the cells with 10 mL of sterile PBS followed by trypsinization (see above). Then, 5 mL of trypsin 0.25% EDTA 1× (Life Technologies Corp. [Gibco], Carlsbad, CA, USA) was added after warming to 37 °C in a water bath. The bottle was incubated for 5 min, and the enzymatic reaction was stopped by adding 5 mL DMEM-EM. Thereafter, the cells were detached from the plastic surface, resuspended in the medium and, after counting, kept in culture or distributed on well plates as required. The medium was changed three times per week. In total, cells from four donors (n = 4) were used for the experiments. These were assigned the numbers 1 to 4. The preparation of the cells was carried out analogously to the previous work of our group [15]. Due to the limited number of donors and cells, we did not have unlimited resources available and therefore could not perform every experiment with the same number of donors. To ensure a sensible distribution of these resources, toxicity analysis was performed with cells from 2 donors, surface characterization with cells from 3 donors, histological staining after stem cell differentiation with cells from 1 donor, RT-qPCR after differentiation with cells from 2 donors, and migration analysis with cells from 3 donors. Additional RT-qPCR experiments were performed with cells from only one donor, which does not allow for a sufficient statistical evaluation of these sub-experiments. These have nevertheless been included in this work for supplementary and descriptive purposes and also for possible future studies. For the toxicity analysis, we used fewer resources than for other experiments, as the corresponding behavior of the cells with regard to cisplatin toxicity was already well documented from previous work, including that of our group. Conversely, in our view, completely omitting a cytotoxicity analysis would also be inappropriate in a study investigating the functionality of MSCs. We performed the histological staining descriptively using cells from only one donor, since we already have RT-qPCR available as an additional method for the purpose of demonstrating the differentiation of MSCs. Furthermore, we believe that a result that can be comparatively presented in diagrams, in which even subtle changes might be observable, is more relevant for interpreting the effect of cisplatin.

### 4.2. Cisplatin Exposures

**Figure 7:**
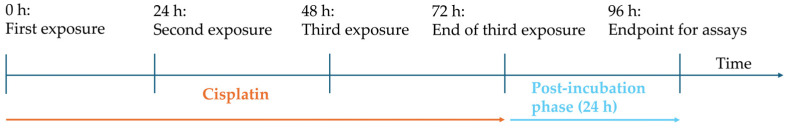
Overview of the exposure assay with cisplatin.

After isolation of MSCs and culture for several days, cells were exposed to cisplatin. The assays were carried out with increasing concentrations of cisplatin (Cisplatin Teva^®^ 1 mg/mL, 3.3 mM, Teva Pharmaceutical Industries Limited, Petach Tikwa, Israel). The cisplatin solution was further diluted with DMEM-EM. The cisplatin concentrations 0.1 μM, 0.5 μM, 1 μM, 2 μM, 5 μM, 10 μM, 20 μM, and 100 μM (as positive control) and a negative sample with DMEM-EM were used for the cytotoxicity evaluation. Our goal in selecting the experimental concentrations for the further experiments was to cover the subtoxic range while still applying a sufficient drug amount to detect any potential effects. Therefore, the experiment to determine the (sub)cytotoxic doses was conducted using a broad range of cisplatin concentrations between 0.1 µM and 100 µM. This corresponds to concentrations of cisplatin that are classically used in drug-based tumor therapy in humans. While the exact dose cannot, of course, be reproduced under these in vitro conditions, a dose should be chosen that comes as close as possible to realistic circumstances. In total, the exposure experiment until the endpoints lasted for 96 h (Figure 7). Cells were exposed at 0 h as follows. Pre-warmed exposure solution (37 °C) was applied, and cells were placed in an incubator with standard cell culture conditions. After 24 h and 48 h, cells were taken out and the exposure solution was aspirated. After 72 h, cells were washed with pre-warmed PBS with Mg^2+^ and Ca^2+^ (PBS^+/+^). Medium without cisplatin was added to all conditions and cells were incubated an additional 24 h prior to analysis at 96 h post first exposure (0 h).

### 4.3. Cytotoxicity Evaluation with the 3-[4,5-Dimethylthiazol-2-yl]-2,5-diphenyltetrazolium-bromid (MTT) Assay

This colorimetric method was used to determine cell metabolic activity as an indicator of cell viability and thus of the cytotoxicity of cisplatin on MSCs. Cells from Donors 2, 3, and 4 (n = 3) were used for this experiment. After exposure to cisplatin as described above, the medium was aspirated. Then, the MTT solution (1 mg/mL in medium; MTT, M5655-100MG, Sigma-Aldrich Chemie GmbH, Steinheim, Germany) was applied with DMEM-EM. Cells were incubated for 4 h, and the solution was aspirated and replaced with 850 μL/well 70% isopropanol (VWR Chemicals, Fontenay-sous-Bois, France). After 30 min incubation at RT in the dark, isopropanol was thoroughly resuspended and 100 μL were transferred to each well in a 96-well plate (6 technical replicates per 850 μL from the original cell culture well) and analyzed at 570 nm in an ELISA microplate reader (ELx800 BioTek Instruments GmbH, Bad Friedrichshall, Germany). Absorbance values were exported to Excel (Excel 2016, Microsoft, Redmond, WA, USA) and the relative viability based on the negative control without cisplatin was calculated.

### 4.4. MSCs Criteria Verification and Evaluation of Cisplatin Impact

The criteria for identification of MSCs according to the “Mesenchymal and Tissue Stem Cell Committee of the International Society for Cellular Therapy” include adherence of the cells, differentiability by induction and histological staining, and specific surface characteristics by flow cytometry [5]. To evaluate these criteria in the cells used in this study, untreated donor cells were analyzed as described below. Furthermore, the cells were treated with different doses of cisplatin and analyzed for the same criteria to evaluate possible effects of cisplatin on differentiation.

#### 4.4.1. Surface Characterization

Flow cytometry was used to examine MSCs with regard to their characteristically expressed surface antigens. MSCs of Donors 2, 3, and 4 (n = 3) were examined for a combination of stromal cell-typical markers CD73, CD90, and CD105 and absence of hematopoietic markers CD31, CD34, and CD45. Flow cytometry was repeated after treatment of the cells with 2 µM and 10 µM cisplatin.

For this purpose, after the MSCs had been detached and counted in the CASY, 2 × 10^5^ cells in DMEM were transferred to a FACS tube (BD 352002, Becton Dickinson GmbH, Heidelberg, Germany), centrifuged at 1400 rpm for 5 min at RT and the supernatant was discarded. One ml PBS with 10% FCS were added to the cell pellet. After resuspension, cells were incubated on ice for 60 min. After centrifugation and decantation, antibodies were added according to the manufacturer’s instructions. After preparation, the samples were analyzed and displayed using the FACSdiva program (BD Bioscience, Heidelberg, Germany). To set fluorescence thresholds, unstained MSCs were used as negative controls to account for autofluorescence. Due to the limited number of donors and cells, no separate isotype checks were performed. Antibodies are listed in Table 1.

#### 4.4.2. Stromal Cell Differentiation and Histological Staining

Different types of differentiation were evaluated using published protocols: (1) osteogenic differentiation, (2) adipogenic differentiation, and (3) chondrogenic differentiation. Cells from Donors 1 and 3 (n = 2) were used for the histological demonstration of differentiation. The procedure was performed without prior treatment with cisplatin as well as after treatment with cisplatin at doses of 2.5 μM, 5 μM, and 10 μM. The experiment was performed in triplicate, and treatment with cisplatin was carried out for 3 consecutive days for 24 h each. Stromal cell differentiation was induced with differentiation additive added DMEM-EM. Cells were seeded in a flask incubated for 24 h, followed by fixation with 4% paraformaldehyde (PFA) in PBS for staining and histological analysis.

For the induction of osteogenic differentiation over 21 days, medium consisting of DMEM-EM with an addition of 50 μg/mL L-ascorbic acid-2-phosphate, 10 mM β-glycerophosphate, and 100 nM dexamethasone was used.

The adipogenic differentiation was performed over 14 days. The medium consisted of DMEM-EM with an addition of 1 μM dexamethasone, 500 μM 3-isobutyl-1-methylxanthine (IBMX), 1 μg/mL insulin, and 100 μM indomethacin.

The chondrogenic differentiation was performed over 21 days. The medium here consisted of DMEM-EM with an addition of 50 μg/mL L-ascorbic acid-2-phosphate, 0.1 μM dexamethasone, 100 μg/mL Pyruvate, 40 μg/mL L-proline, 1% ITS^+^1, and 10 ng/mL TGF-β.

Histological staining was performed after washing with distilled water and rehydration.

For staining to demonstrate osteogenic differentiation through calcium detection according to Von Kossa, the covering was carried out with a 1% silver nitrate solution (Sigma-Aldrich Chemie GmbH, Steinheim, Germany) and irradiation with a UV lamp (Desaga Uvis, Desaga GmbH, Heidelberg, Germany) for 20 min. After washing, the addition of 5% sodium thiosulfate solution (Merck KGaA, Darmstadt, Germany), and washing again, the cell nuclei were stained with Nuclear Fast Red Solution (1001210500, Sigma-Aldrich Chemie GmbH, Steinheim, Germany). After washing with distilled water, the cells were dried with ethanol.

A further calcium deposit staining of the cells was carried out after washing with Alizarin Red S solution (TMS-008-C, Merck KGaA, Darmstadt, Germany). Here, water-insoluble red–orange salts were formed by reaction with calcium deposits in the cells.

With the Oil Red O stain, fat vacuoles were detected as a sign of adipogenic differentiation. After washing, the treatment was carried out with 100% 1,2-propanediol (propylene glycol, P4347-500ML, Sigma-Aldrich Chemie GmbH, Steinheim, Germany) for 5 min. After suction, the cells were incubated with Oil Red O (Sigma-Aldrich Chemie GmbH) previously heated to 60 °C and incubated at 60 °C for 10 min. Afterwards, the cells were treated with 85% 1,2-propanediol for 1 min. The cells were then washed with distilled water, which was repeated three times. Finally, the cell nuclei were stained with hematoxylin solution (1051740500, Merck KGaA, Darmstadt, Germany) for 30 s.

Chondrogenic differentiation was visualized with Alcian Blue staining, which stains acidic mucopolysaccharides, including proteoglycans and glycosaminoglycans, which are abundant in cartilage. After washing, the sections were treated with 3% glacial acetic acid for 3 min. Subsequently, Alcian Blue solution was applied. After treatment with Nuclear Fast Red and repeated washing before and after, dehydration was performed using an ascending EtOH series. The sections were then dried and mounted with Entellan.

#### 4.4.3. Real-Time Quantitative Polymerase Chain Reaction (RT-qPCR)

mRNA abundance was quantified by RT-qPCR. Cells from Donors 1 and 3 (n = 2) without prior treatment with cisplatin, as well as after treatment with cisplatin at doses of 2 and 10 μM, were used for this experiment. Furthermore, the experiment was additionally carried out with 2 μM cisplatin with one donor and 10 μM cisplatin with another donor and a 5-day (instead of 3-day) incubation period with cisplatin in order to additionally receive indications for a possible effect of an additional temporal factor. The 5-day incubation was performed the same as for the 3-day protocol with new medium, with or without cisplatin, for 5 days.

Gene expression was quantified by RT-qPCR. Cells from 2 donors (n = 2) were used for this experiment. After detaching the cells and counting, 2 × 10^5^ cells/well were seeded in duplicates. Plates were treated with cisplatin concentrations of 2 μM and 10 μM and a negative control with DMEM-EM for 24 h. The Quiagen RNeasy Kit (Qiagen GmbH, Hilden, Germany) was used for RNA isolation. A photometric RNA content measurement was carried out, for which 79 μL DEPC-H_2_O (Sigma-Aldrich Chemie GmbH, Steinheim, Germany) were placed in an UVette (Eppendorf AG, Hamburg, Germany), 1 μL of the RNA solution was added, the content was analyzed in a photometer (Eppendorf AG, Hamburg, Germany), and 80 μL DEPC-H_2_O served as a reference. RNA samples were then frozen at −80 °C until beginning of analysis.

To synthesize cDNA from RNA, samples were thawed at room temperature. For each cisplatin concentration, 3 wells were equipped in a reaction vessel (Fast Reaction Tubes, Micro Amp 8 Cap strips, Life Technologies Corp. [Applied Biosystems], Carlsbad, CA, USA). Quantities of 50 ng/well of RNA, 4 μL of Superscript (SuperScript^®^ VILO Mastermix Taq Man Gene Expression Master Mix, Life Technologies Corp. [Applied Biosystems], Carlsbad, CA, USA) and DEPC-H_2_O were pipetted to obtain a volume of 20 μL/well. The vessel was sealed with adhesive foil (Sarstedt AG & Co. KG, Nümbrecht, Germany) and centrifuged at 1300 rpm for 3 min. For cDNA synthesis, the vessel was placed in the RT-qPCR cycler (StepOnePlus Real-Time PCR System, Life Technologies Corp. [Applied Biosystems], Carlsbad, CA, USA). This resulted in a double-stranded cDNA. After the program, the finished cDNA was transferred to Eppi tubes and frozen at −80 °C.

TaqMan probes and GAPDH as a reference gene were used. Three wells were created for each cisplatin concentration per probe on the 96-well plate (Micro Amp Fastoptical 96-Well Plate, Applied Biosystems, life technologies GmbH, Darmstadt, Germany). For each assay, the mixture was prepared in 1.5 mL Eppi tubes, which contained 10 μL master mix, 7 μL DEPC-H_2_O, and 1 μL of the corresponding assay per well. Quantities of 18 μL/reaction of the mixture and 2 μL/reaction of the respective cDNA were mixed, and the plate was provided with an adhesive film, centrifuged at 860 rpm for 5 min, and placed in the RT-qPCR cycler. After incubation at 95 °C for 10 min, 40 replication cycles were run and, after completion, reached the CT value for each sample, from which the relative content (fold change) could be calculated.

### 4.5. Scratch Assay for Migration Analysis

In this assay, an artificial wound is created in the sense of a cell-free aisle. The wound area (in %) is then observed at 0, 24, and 96 h. Cells from Donors 2, 3, and 4 (n = 3) were used for this experiment. After transferring 8 × 10^4^ cells/well into a 12-well plate, treatment with 2 μM cisplatin and negative controls (DMEM-EM) were carried out. A vertical “scratch” was carried out in each well: a sterile 1 mL pipette tip was used to scratch an artificial “wound” (cell-free area) at the center of each well. After scratching, the detached cells were removed by resuspending and aspirating the medium. One mL/well PBS was added, and microscopic images at 4× and 10× magnification were recorded (automated, inverse transmitted light microscope LEICA DMI 4000 B Leica Microsystems GmbH, Wetzlar, Germany; microscope control box LEICA CTR 4000, Leica Microsystems GmbH, Wetzlar, Germany; software LAS V4.8, Leica Microsystems GmbH, Wetzlar, Germany). PBS was then aspirated, 1 mL DMEM-EM was added, and cells were incubated at 37 °C for 24 h, and the imaging procedure was repeated after 24 h and 96 h. The image analysis of “wound closure” was carried out with ImageJ (1.53k, Rasband, W.S., U.S. National Institutes of Health, Bethesda, MD, USA), reporting a measure of cell-free area for each image [34].

### 4.6. Illustration of Data and Statistical Evaluation

GraphPad Prism software version 9 (GraphPad Software, Inc., La Jolla, CA, USA) was used for statistical analysis. One-way ANOVA and Dunnett’s tests were used to test for statistical differences in treated samples in comparison with the negative controls of the MTT assay, the flow cytometry, the RT-qPCR, and the scratch assay. A *p*-value of *p* ≤ 0.05 was set as the level of significance (marked with “*” in plots).

## Figures and Tables

**Table 1:** Antibodies used for surface characterization.

Antibody	Source
APC Mouse Anti-Human CD90 (559689)	Becton Dickinson GmbH, Heidelberg, Germany
FITC Mouse Anti-Human CD105 (555690)	Becton Dickinson GmbH, Heidelberg, Germany
FITC Mouse Anti-Human CD45 (555482)	Becton Dickinson GmbH, Heidelberg, Germany
PE Mouse Anti-Human CD31 (555446)	Becton Dickinson GmbH, Heidelberg, Germany
PE Mouse Anti-Human CD34 (550761)	Becton Dickinson GmbH, Heidelberg, Germany
PE Mouse Anti-Human CD73 (550257)	Becton Dickinson GmbH, Heidelberg, Germany
FITC, Alexa Fluor^®^ 488 Goat anti-Mouse IgG, 1:1000	Life Technologies Corporation, Carlsbad, CA, USA

## Data Availability

The original contributions presented in this study are included in the article. Further inquiries can be directed to the corresponding author.

## Abbreviations

The following abbreviations are used in this manuscript:

ADSCs	Adipose tissue-derived mesenchymal stem cells
BM-MSCs	Bone marrow-derived multipotent mesenchymal-stromal cells
°C	Degrees Celsius
cDNA	Complementary DNA
CD	Cluster of differentiation
cisplatin	Cis-diamminedichloroplatinum (II)
cm^2^	Square centimeter
DMEM-EM	Dulbecco’s Modified Eagle Medium—Expansion Medium
DNA	Deoxyribonucleic acid
e.g.,	Exempli gratia (for example)
ELISA	Enzyme-linked immunosorbent assays
EtOH	Ethanol
FCS	Fetal calf serum
GAPDH	Glyceraldehyde-3-phosphate dehydrogenase
h	Hours
IBMX	3-isobutyl-1-methylxanthine
IDO1	Indoleamine 2,3-dioxygenase 1
LEP	Leptin
MDSCs	Myeloid-derived suppressor cells
min	Minutes
mL	Milliliter
mM	Millimolar
mRNA	Messenger RNA
MSCs	Mesenchymal stromal cells
MTT	3-[4,5 Dimethylthiazol-2-yl]-2,5-diphenyltetrazolium-bromid
Neg.	Negative control
nM	Nanomolar
ns	Non-significant
PBMCs	Peripheral blood mononuclear cells
PBS	Phosphate-buffered saline
PFA	Paraformaldehyde
RNA	Ribonucleic acid
rpm	Revolutions per minute
RT	Room temperature
RT-qPCR	Real-time quantitative PCR
RUNX2	Runt-related transcription factor 2
SOX9	SRY-box transcription factor 9
TDLNs	Tumor-draining lymph nodes
TGF-β	Transforming growth factor beta
TSG-6	Tumor necrosis factor-stimulated gene-6
UV	Ultraviolet
μM	Micromolar
μL	Microliter
